# 

**Published:** 1849-06

**Authors:** 


### INDEX TO VOL. III.

[THIRD SERIES.]

A

Agassiz's Zoology .................. 53
Albany Medical College ..........182
Alkalies in Rheumatism...........266
Amenorrhea ........................116
American Medical Association.....541
Anatomy, Morton's ................332
Arsenic, poisoning by ............267
Auscultation. Barth & Roger on...55
Antiaphrodisiac, sugar as an .....451

B

Barnett on cholera................387
Belladonna as a preventive of scarlatina...........................118
Bell on cholera.........303, 398, 510
Bismuth in diarrhea of phthisis ...343
Bladder, puncture of..............530
Brachet's lectures ..............93, 487
Brain improved by disease ........272
Brockett on a Fever in Tennessee ..........................199
Buchanan on cholera..............369

C

Calomel, poisoning by ..............84
Camphor and chloroform mixture.432
Carbonic acid exhaled, quantity of..537
Castor oil in typhoid fever .......544
Chloride of zinc, antidote for ....434
Chloroform, death from......258, 459
Chloroform, discoverer of ........179
Chloroform in dysmenorrhea.....113
Chloroform in mania..............447
Chloroform in tetanus ........89, 120
Chloroform in cholera .......119, 170
Chloroform in labor ..............544
Chloroform in various diseases...340
Chloroform in lumbago ..........525
Chloroform in mania-a-potu.......540
Chloroform, Warren on...........455
Cholera, Lawson on ...............91
Cholera, Sutherland on ..........260
Cholera, progress of.....90, 161, 162
.........................166, 262, 547
Cholera in New York .............133
Cholera in granite regions ..169, 178
Cholera, vapor-baths in ..........170
Cholera in Camden, Arkansas ...387
Cholera in Clarksville.............379
Cholera in Nashville ..............369
Cholera in N. Orleans..141, 245, 363
Cholera in Louisville ........144, 5[illegible]
Cholera, Bell on .......303, 398, 51[illegible]
Cholera, Haskins on ..............379
Cholera, synopsis of treatment...337
Cholera, Paris committee on......521
Cholera, calomel in ...............533
Cholera, treatment of .............547
Cod-liver oil........................535
Collodion as a coating for pills ...452
Collodion in toothache ............259
Collodion in diseases of the skin..420
Colic ..................................37
Cooper, Samuel death of..........276
Cotton's case of tetanus...........204
Cream tartar, adulteration of ....452
Croup, Green on ..................122
Croup, membranous ..............159
Croup, iodine in...................540
Cryptogamous origin of fevers...443

D

Dawson on diseases of Ohio ......462
Death from chloroform ......358, 459
Diseases of advanced life .........390
Drake, resignation of..............365
Rromgoole's case of colic .........37
Dysentery, nit. silver in ..........451
Dysmenorrhea, chloroform in ....118

E

Editorial changes...................274
Erysipelas, kreosote in............636
Etherization .........................1

Eve's introductory lecture ........49
Eve's, P. F. case of Lithotomy...439

F

Febrifuge, a new one ............83
Fee on typhoid fever ............185
Fevers, typhus and typhoid.......67
Fevers in Alabama ...............90
Fevers in Tennessee ............199
Fevers in West Indies, quinine ..253
Fownes, death of ...............451
Fracture tables ................457

G

Galium tinctorum in diarrhea....206
Galvanism in paralysis of bladder .539

H

Hamilton's fracture-tables ........457
Harbert's case of extra-uterine pregnancy ....................110
Haskins on cholera..............379
Hood's principles of medicine ....40
Human parturition, Miller on....219
Hydrocephalus, congenital ........105
Hydrocephalus and hypertrophy of the brain ..................175

I

Influenza ........................92
Insanity, Wible's case of ........289
Iodine in croup .................540

K

Kirtland's introductory lecture ....47
Kreosote in erysipelas............536

L

Labor, with flooding ............287
Lecture-term, extension of ........83
Lipscomb on moles. ..............277
Lithotomy ......................439
Lithotomy and lithotrity compared ......................449
Liver, sugar in .................260
Louisville, University of.........454
Louisville, cholera in .......144, 510
Lunatic hospitals...............458

M

Maddin's case of tetanus ........120
Mania, chloroform in ............447
Medicine, sectional ...............89
Medical schools ..................85
Meig's introductory lecture ........49
Meig's obstetrics ................548
Meningo-cerebritis ..............202
Merrick's introductory lecture....181
Meteorology and health ..........438
Miller's midwifery, review of....219
Miller's, W. H. anomalous case...495
Moles, cause of..................277
Mortality of N. York in 1848....455
Mulder's counterblast to the potato ........................176

N

Naphtha in diarrhea..............524
Nitrate of silver in dysentery ....451
Nocturnal visits.................540
Norris's introductory lecture .....180

O

Ohio, diseases of................562
Opium, muriate of................258
Orfila, anecdote of ..............366
Osborne's case of meningo-cerebritis ........................292
Osborne's notice of Galium-tinctorum ........................206

P

Paris, medical profession in ......84
Paralysis of bladder .............539
Penis, fracture of................536
Personal portraits ...............275
Pills coated by collodion ........452
Poisoning by bad calomel .........84
Poisoning by arsenic.............267
Poisoning by chloride of zinc ...434
Potato, Mulder on ...............176
Pregnancy, extra-uterine..........110
Prentis's case of hydrocephalus...105
Presence of sugar in liver.......260
Prichard, death of ..............457
Puerperal fever, quinine in......344

Q

Quinine rendered tasteless.......179
Quinine in fevers of W. Indies..253
Quinine a prophylactic in puerperal fever .....................344
Quinine, Scruggs on.............344
Quinine in tetanus...............357

R

Rain in New Orleans in 1848....538
Rectum in a cul-de-sac ..........546
Rheumatism ....................193

S

Ship-fever, Smith on............347
Small-pox, Vigo's plaster for.....539
Smith's introductory lecture......181
Southworth on typhoid fever ....208
Strother's case of cholera........119
Sugar in the liver ...............260
Sugar as an antiaphrodisiac .....451
Sutherland on the treatment of cholera.......................260

T

Tennessee, fever in..............199
Tetanus................89, 120, 357
Toothache, colodion and asbestos in......................259
Tumors, sudden development of..204
Typhoid fever, Fee on...........185
Typhoid fever, Flint's case......171
Typhoid fever, Brachet on...93, 487
Typhus and typhoid fevers........67
Typhus and typhoid fevers in Kentucky and Missouri.........208

U

University of Louisville...........454
Uterus, neuralgia of..............449
Uterine hemorrhage..............287

V

Valedictory addresses.............459
Vaughan on vegetation...........183
Vegetation, cause of.............183
Vigo's plaster in small-pox.......539

W

Wendel's hospital reports.........193
Wible's case of insanity...........289
Wilcox's case of amenorrhea.....116
Wither's case of dysmenorrhea...113

Y

Yandell, D. W. on pleuro-pneumonia..........................209
Yandell, L. P., on etherization......1
Yandell's L. P. case of uterine hemorrhage......................287

Z

Zoology, Agassiz and Gould's.....53
Zagorsky, death of...............449

To Subscribers.—A volume of this Journal ends with the present number, and it is a fit occasion to call the attention of subscribers to the necessity of remitting. They will see that our receipts are extremely meager; but there is no intermission in the expenditures. We trust, gentlemen, that you will favor us without further delay.
PRENTICE & WEISSINGER.

RECEIPTS OF MEDICAL JOURNAL FOR MAY, 1849.

| | |
|---|---|
| Dr. A. D. Guthrie, Trinity Springs, Ind., | $5 00 |
| J. H. Donnell, Franklin, Ind., | 5 00 |
| —— Rose, Gallatin, Mo. | 5 00 |
| J. W. Holmes, McAllister's ⋈ Roads, Tenn. | 5 00 |
| G. W. Wheeler, Perryville, Mo. | 5 00 |

The Western Journal of Medicine and Surgery is published monthly by the undersigned, at the corner of Main and Fifth streets, Louisville, at $5 per annum, payable in advance. Each number contains 96 pages, making two volumes in the year of more than 550 pages each.

Contributions to its pages by the Physicians of the Valley of the Mississippi, addressed to the Editors, are respectfully solicited.

Letters on business, to be addressed, postage paid, to the publishers.

January, 1844. PRENTICE & WEISSINGER.

